# Measuring Prospective Imagery: Psychometric Properties of the Chinese Version of the Prospective Imagery Task

**DOI:** 10.3389/fpsyg.2021.645127

**Published:** 2021-05-25

**Authors:** Mingfan Liu, Yiting Chen, Xiaoying Yin, Dandan Peng, Xinqiang Wang, Baojuan Ye

**Affiliations:** ^1^Center of Mental Health Education and Research, Jiangxi Normal University, Nanchang, China; ^2^Institute of Psychological Technology Application, Jiangxi Normal University, Nanchang, China; ^3^Jiangxi Provincial People’s Hospital, Nanchang, China

**Keywords:** Prospective Imagery Task, Chinese version, validity, reliability, depression

## Abstract

**Objective:**

Prospective negative imagery is suggested to play an important role in the development and maintenance of anxiety and depression. The Prospective Imagery Task (PIT) was developed to assess prospective imagery. Given the importance of prospective imagery for mental health in the Chinese cultural context, our objective was to examine the psychometric properties of the PIT in a Chinese sample.

**Methods:**

The instrument was validated among a sample of 1,372 Chinese individuals (mean age = 19.98, *SD* = 4.57; 35.2% male) who completed the PIT immediately following the Beck Depression Inventory-II (BDI-II) and State-Trait Anxiety Inventory-Trait version (STAI-T).

**Results:**

The two-factor structure of the PIT was in line with the original study, with satisfactory reliability and positive correlations with the BDI-II and STAI-T scores. Latent profile analysis revealed a three-class pattern. The measurement invariance indicated that the instrument can be used among different age groups as well as among males and females.

**Conclusion:**

The Chinese version of the PIT is a reliable and valid tool to measure prospective imagery, and the positive subscale is meaningful for clinical psychology. Limitations and future research directions are discussed.

## Introduction

Mental imagery differs from real perception; the former is the perceptual experience of individuals without parallel sensory input (Kosslyn et al., 2006) and the simulation or recreation of perceptual experience (Kosslyn et al., 2001). Previous experimental cognitive studies have focused on non-emotional mental imagery. However, in the past 20 years, emotional mental imagery has received increasing attention. Holmes and Mathews (2010) emphasized a “special relationship” between mental imagery and emotions. Mental imagery has been viewed as an emotional amplifier or a potent driver of emotion (Burnett Heyes et al., 2013).

Research in clinical and counseling psychology has focused on the mental imagery of emotional disorders in particular. A growing number of studies have found a link between imagery and emotional disorders (Brewin et al., 2010). Blackwell et al. (2013) found a negative correlation between depression and the vividness of imagining positive future events, and Werner-Seidler and Moulds (2011) suggested that people with depression have impairments in their ability to visualize the future. Compared with healthy people, people with depression and anxiety have lower subjective estimates of the vividness and likelihood of positive future events (Morina et al., 2011), and people with bipolar disorder have more vivid imagery of negative future events (Holmes et al., 2011). From a clinical perspective, stimulating future events through imagery is important if the action is negative (Holmes et al., 2007); for example, some people with depression have intrusive mental images of future suicide (Crane et al., 2012).

Tool has been generated to measure the vividness of imagery for prospective events. Initially, Stöber (2000) investigated the vividness of prospective positive and negative mental imagery among a non-clinical population using the 30-item Prospective Imagery Task (PIT, based on MacLeod and Byrne, 1996) on a 7-point scale. The scale was proven to have satisfactory reliability and validity. Based on Stöber’s (2000) PIT, Holmes et al. (2008) shortened the instrument to 20 items and measured it on a 5-point scale; they reported that its reliability and validity were in line with Stöber’s (2000) findings.

The advantage of the PIT is that it has been widely used in many studies related to mental imagery (Morina et al., 2011; Blackwell et al., 2013). Therefore, the PIT can be replicated by comparison with previous studies and compared among a variety of countries. This is an advantage because Chinese culture is quite different from that of the West, and the generation and content of imagery are closely related to culture. Because culture is a vital element in mental imagery, cross-cultural validation of the PIT is necessary (Yoon et al., 2016). For instance, a study found that culture, as opposed to the language of a message, drove imagery-generation capabilities among participants from China, Singapore, and the United States (Liang et al., 2010). When exposed to abstract advertising messages, East Asians tend to generate more imagery than Westerners (Liang and Kale, 2012). In addition to cultural differences, mental imagery is related to various emotional disorders. A decrease in the expectation of future positive events is a typical feature of depression, while anxiety is characterized by an increase in the number of perceived negative future events (Rief et al., 2015; Gadassi Polack et al., 2020).

Thus, the present study attempted to establish the criterion validity of the PIT by exploring its correlation with depression as measured by the Beck Depression Inventory-II (BDI-II) (Beck et al., 1996) and with anxiety as measured by the State-Trait Anxiety Inventory-Trait version (STAI-T) (Spielberger et al., 1970).

## Materials and Methods

### Participants and Procedure

A total of 1,500 participants were recruited. Of these participants, 128 were excluded because (a) they were younger than 16 or older than 65, (b) they were unwilling to participate/give informed consent, or (c) they had a history of psychiatric illness. Therefore, the effective sample comprised 1,372 participants. Of these, 483 (35.2%) were male, and 889 (64.8%) were female. Students constituted the majority of the participants (83.5%). The mean age of the overall sample was 19.98 years (*SD* = 4.57).

Questionnaires were distributed online and offline. The study was approved by the institute’s ethics committee. Written informed consent was obtained from the participants after they received a description of the study. Teenage participants obtained informed consent from their guardian. Each participant received a reward of 5 yuan.

The participants were administered questionnaires twice. At baseline (i.e., the pretest), the sociodemographic and clinical characteristics, including age, gender and career, of the 1,372 participants were collected together with three self-report scales, including the PIT, BDI-II, and STAI-T. At 2 months following the pretest, the PIT was sent to 60 of the participants online to assess the test-retest reliability of the scale.

### Measures

#### Prospective Imagery

Prospective imagery was measured with the PIT containing 10 positive (e.g., “People you meet will like you”) and 10 negative (“You will be the victim of crime”) future scenarios (Holmes et al., 2008). The Chinese translation of the PIT was developed through iterative back-translation by a team of bilingual psychologists and with the help of one of the authors. Consequently, we modified the translation by comparing its comprehension and accuracy with the original PIT and determined the final version. Participants were asked to rate the vividness of each image on a 5-point scale ranging from 1 (“no image at all”) to 5 (“very vivid”). A higher score for each subscale (positive or negative) indicates more vivid imagery. The PIT has good internal consistency (0.83 < α < 0.90) (Stöber, 2000; Blackwell et al., 2013).

#### Depression

We assessed depression with the Chinese version of the Beck Depression Inventory-II (BDI-II) (Wang et al., 2011), a 21-item self-report scale. Participants were asked to rate each item on a 4-point scale ranging from 0 (“rarely or none of the time”) to 3 (“most or all of the time”). The higher the score, the more severe the level of depression. The Chinese version of the BDI-II was found to have good reliability and validity among Chinese populations (Wang et al., 2011).

#### Anxiety

The Chinese version of the State-Trait Anxiety Inventory-Trait version (STAI-T) (Shek, 1988) was used to measure trait anxiety. The inventory consists of 20 anxiety-related items. Participants were asked to rate how they “generally feel” on a 4-point scale. Two previous studies tested and validated the Chinese version of the STAI-T for use in the Chinese community with good reliability (Shek, 1988) and validity (Shek, 1993).

### Data Analysis

The data set of the participants was randomly divided into two halves to explore factor structure. Exploratory factor analysis (EFA) was conducted with half of the sample (*n* = 686), and the maximum likelihood robust estimator (MLR) method was used to extract the factor loadings. The other half of the sample (*n* = 686) was used for confirmatory factor analysis (CFA) with the maximum likelihood (ML) method. Goodness-of-fit indices were reported, including the Tucker-Lewis index (TLI), comparative fit index (CFI), Akaike information criterion (AIC), Bayesian information criterion (BIC), root mean square error of approximation (RMSEA), and standardized root mean square residual (SRMR). TLI and CFI range in value from zero to 1.00, with a value close to 1.00 indicating a better fit (Mulaik et al., 1989). For RMSEA and SRMR, values less than 0.05 are considered good, values less than 0.08 are appropriate, and values greater than 0.10 indicate that there is room for improvement (Finch and West, 1997).

We assessed the internal consistency of the PIT using Cronbach’s alpha coefficients and test–retest reliability with Pearson’s correlation coefficient. According to Cicchetti (1994), values less than 0.60 are poor, values between 0.70 and 0.80 are acceptable, and values greater than 0.80 indicate good reliability.

To examine validity, Pearson’s correlation coefficients of the PIT, BDI-II, and STAI-T were examined. Then, regression analyses were conducted to examine whether prospective imagery contributed independently to the prediction of depression and anxiety after adjustment for gender, age, and career. In the first step, the two facets of prospective imagery were entered into the regression model. In the second step, gender, age and career were entered.

All receiver operating characteristic (ROC) curve analyses were conducted using SPSS 23.0. For each ROC curve analysis, we calculated the area under the ROC curve (AUC) (Green, 1989) and the optimum cut-off point (Youden index) to distinguish individuals with and without depression or anxiety and to determine the optimal cut-off point to maximize sensitivity and specificity. The critical value for significance for AUC was set at *p* = 0.05. Šimundić (2009) suggested that an AUC value greater than 0.80 is excellent, 0.70–0.80 is good, 0.60–0.70 is sufficient, and less than 0.60 is poor.

We conducted latent profile analysis (LPA) for the vividness of positive and negative prospective imagery using maximum likelihood estimation with robust standard errors, judging the latent category and distribution in Mplus 7.11 (Muthén and Muthén, 1998–2015). We gradually increased the number of types of LPA; the smaller the model fitting index of AIC, BIC, and aBIC, the better the model fit. The value of entropy represents classification accuracy, and its general criterion is 0.80 (Clark, 2010). Higher entropy and significance levels of the Lo–Mendell–Rubin (LMR) test and bootstrapped likelihood ratio test (BLRT) (*p* < 0.05) indicate that the model of k categories is better than the model of k - 1.

Mplus 7.11 was also used to examine the measurement invariance (MI) across gender and age by means of multigroup CFA. Since MI compares a series of nested models, in addition to the commonly used fitting indexes, such as χ^2^, CFI and RMSEA, we can use Δχ^2^. Nevertheless, in large sample cases, compared with Δχ^2^, ΔCFI and ΔRMSEA are superior for evaluating model fit (ΔCFI < 0.01, ΔRMSEA < 0.015) (Finch and French, 2018; Counsell et al., 2020). Due to the large sample size, the MI in our study was mainly examined through ΔCFI and ΔRMSEA.

Data collected at baseline with a total sample of 1,372 participants were used to estimate the internal consistency using Cronbach’s α coefficients. Pearson’s correlation coefficients between the PIT scores at baseline and the 2-month follow-up were calculated to examine the test–retest reliability. To validate the Chinese version, its correlations with the BDI-II and STAI-T were examined. We conducted regression analyses to examine whether prospective imagery dependently predicted depression and anxiety. LPA was used to determine the optimal number of latent profiles. The MI was used to test the general applicability of the PIT.

## Results

### Factor Structure

Through EFA, we examined the potential factor structure of the PIT. According to Kline (2010), compared with the single-factor model, the AIC, and BIC of two-factor model decreased the most sharply. Integrating other fitting indexes, the two-factor model was the best (see Table 1, χ^2^/*df* = 2.68, TLI = 0.90, CFI = 0.91, RMSEA = 0.05, SRMR = 0.04). The factor loadings for each item are illustrated in Table 2. All items that loaded on Factor 1 originally belonged to the negative subscale, except for Item 18, which originally belonged to the positive subscale. All items that loaded on the second factor belonged to the positive subscale. The factor loadings of all items were greater than 0.40. The correlation between the two factors was small (*r* = 0.27).

**TABLE 1:** The goodness-of-fit indices of the factor analysis models.

Model	χ ^2^	*df*	χ ^2^/*df*	TLI	CFI	AIC	BIC	RMSEA	SRMR
Single-factor model(EFA)	1491.07	170	8.77	0.54	0.48	43355.28	43627.13	0.11	0.12
Two-factor model(EFA)	404.91	151	2.68	0.90	0.91	42166.91	42524.56	0.05	0.04
Three-factor model(EFA)	413.98	133	3.11	0.86	0.90	42063.88	42503.66	0.06	0.03
Two-factor model(CFA)	589.14	169	3.49	0.86	0.88	42092.01	42368.39	0.06	0.06

**n* = *686. TLI*, *Tucker-Lewis index; CFI*, *comparative fit index; AIC*, *Akaike information criterion; BIC*, *Bayesian information criterion; RMSEA*, *root mean square error of approximation; SRMR*, *standardized root mean square residual.**

**TABLE 2:** Factor loadings extracted by factor analysis with oblique rotation.

Item No.	Item	Subscale	Factor 1	Factor 2
1	You will have a serious disagreement with a good friend	Negative	0.41	
2	People will admire you	Positive		0.54
3	You will have health problems	Negative	0.53	
4	You will make a decision you regret	Negative	0.45	
5	You will feel misunderstood	Negative	0.51	
6	You will have lots of energy and enthusiasm	Positive		0.58
7	You will do well in your course	Positive		0.64
8	You will get the blame for things going wrong	Negative	0.46	
9	You will achieve the things you set out to do	Positive		0.56
10	You will be the victim of crime	Negative	0.51	
11	Someone close to you will reject you	Negative	0.67	
12	Things won’t work out as you had hoped	Negative	0.65	
13	People will dislike you	Negative	0.76	
14	You will be very fit and healthy	Positive		0.54
15	People will find you dull and boring	Negative	0.58	
16	You will have lots of good times with friends	Positive		0.60
17	You will be able to cope easily with pressure	Positive		0.54
18	You mind will be very alert and “on the ball”	Positive	0.42	
19	You will make good and lasting friendships	Positive		0.62
20	People you meet will like you	Positive		0.50

**n* = *686. Only factor loadings greater than 0.40 are presented.**

Based on the two-factor model obtained by EFA, CFA was performed on the other half of the sample data (*n* = 686, see Table 1). Subsequent analyses of reliability and criterion validity were based on the proposed structure of the correlated two-factor model.

### Reliability

Cronbach’s alpha coefficients of the total scale and subscales were as follows: total PIT (Cronbach’s α = 0.84), positive (Cronbach’s α = 0.81), and negative (Cronbach’s α = 0.83). All items were substantially linearly correlated with the underlying construct they were intended to measure (i.e., corrected item-scale correlation was 0.55 or greater).

The test-retest reliability for the total scale and subscales was as follows: total PIT *r* = 0.89 (*p* < 0.01), positive *r* = 0.78 (*p* < 0.01), and negative *r* = 0.89 (*p* < 0.01).

### Validity

As shown in Table 3, positive and significant correlations were found among depression, anxiety, and negative prospective imagery, whereas negative and significant correlations were found among depression, anxiety, and positive prospective imagery. Both subscales were related to depression and anxiety even after controlling for differences in gender, age and career (*p* < 0.001, Δ*R*^2^ = 0.03, see Table 4).

**TABLE 3:** Correlation matrix of PIT, BDI-II, and STAI-T.

	1	2	3	4	5
1. BDI-II	–				
2. STAI-T	0.74**	–			
3. PIT-P	−0.30**	−0.36**	–		
4. PIT-N	0.31**	0.30**	0.27**	–	
5. PIT-Total	0.04	0.00	0.75**	0.84**	–

*N* = 1,372. **p < *0.01. PIT, Prospective Imagery Task; BDI-II, Beck Depression Inventory-II; STAI-T, State-Trait Anxiety Inventory-Trait version; PIT-P*, *Prospective Imagery Task-Positive; PIT-N*, *Prospective Imagery Task-Negative.*

**TABLE 4:** Results of regression analyses showing prediction of depression (BDI-II) and anxiety (STAI-T) by prospective imagery (PIT).

	Depression		Anxiety	
	β	Sig.	β	Sig.
**Step 1**				
PIT-P	−0.42	0.000	−0.47	0.000
PIT-N	0.42	0.000	0.42	0.000
*R*^2^	0.25		0.29	
**Step 2**				
PIT-P	−0.40	0.000	−0.46	0.000
PIT-N	0.42	0.000	0.42	0.000
*R*^2^	0.28		0.32	

**N* = *1,372. PIT, Prospective Imagery Task; BDI-II, Beck Depression Inventory-II; STAI-T, State-Trait Anxiety Inventory-Trait version; PIT-P*, *Prospective Imagery Task-Positive; PIT-N*, *Prospective Imagery Task-Negative.**

### ROC Curve Analysis

An optimal cut-off point is valuable for discriminating between clinical and healthy populations. Thus, cut-off points for the PIT were examined by using the recommended cut-off of 28 on the BDI-II and 48 on the STAI-T. As shown in Tables 5, 6, when we took the BDI-II and STAI-T scores as the state variables, only the AUC value of the PIT-P for BDI-II was fair (AUC = 0.80, *p* < 0.05).

**TABLE 5:** Area under the curve (AUC) of PIT.

		Area	Std. error	Sig.	Asymptotic 95% confidence interval	
					Lower bound	Upper bound
BDI-II	PIT-P	**0.80**	0.16	0.039	0.49	1.00
	PIT-N	0.20	0.11	0.030	0.00	0.40
STAI-T	PIT-P	0.65	0.02	0.000	0.62	0.68
	PIT-N	0.36	0.02	0.000	0.34	0.39

**N* = *1,372. PIT, Prospective Imagery Task; BDI-II, Beck Depression Inventory-II; STAI-T, State-Trait Anxiety Inventory-Trait version; PIT-P*, *Prospective Imagery Task-Positive; PIT-N*, *Prospective Imagery Task-Negative.* Bold indicates AUC value was fair.*

**TABLE 6:** ROC curve coordinate point of PIT-P (part).

	Diagnostic point	Sensitivity	1—Specificity	Youden index
BDI-II	18.50	0.95	0.25	0.70
	19.50	0.93	0.25	0.68
	20.50	0.91	0.25	0.65

**BDI-II, Beck Depression Inventory-II; PIT-P*, *Prospective Imagery Task-Positive.**

In ROC curve analysis, the Youden index (sensitivity + specificity − 1) is often used to represent the cut-off point for maximum discrimination. As shown in Table 6, when the BDI-II score was taken as the state variable, the optimum screening score was 19 (sensitivity = 94.8%, specificity = 75.0%, Youden index = 0.70).

### Latent Profile Analysis

**FIGURE 1:**
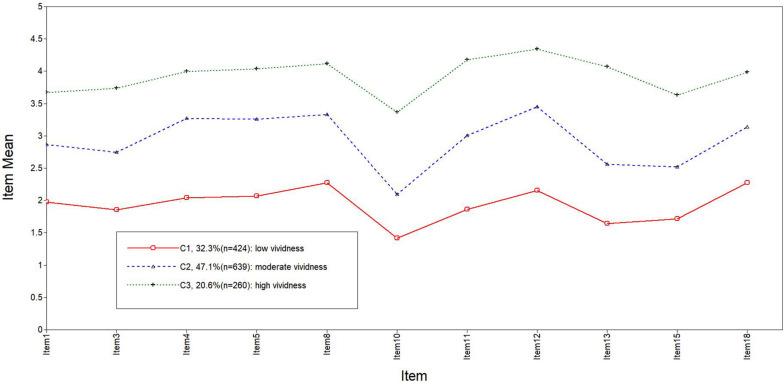
Plot of the standardized mean scores on the positive subscale across the three latent profiles.

**FIGURE 2:**
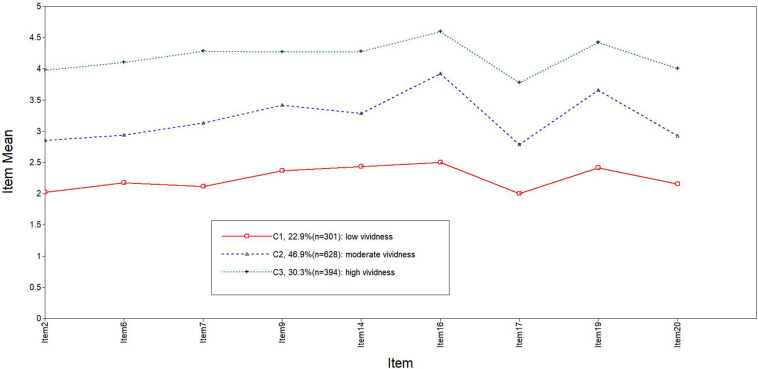
Plot of the standardized mean scores on the negative subscale across the three latent profiles.

The PIT model fitting indices are shown in Table 7. Referring to Nylund et al. (2007), LPA was conducted by starting with two types and gradually increasing the number of types. The fitting indices in the class 3 model exhibited the sharpest fall, and this model was simpler than the others. Therefore, the class 3 model is the best model. The score distribution of the latent class of prospective imagery on each item is shown in Figures 1, 2. The population of class 1, which accounted for 22.9 and 32.3% of positive and negative prospective imagery, respectively, was named the “low vividness” group (C1). Class 2 accounted for 46.9 and 47.1%, respectively, and was named the “moderate vividness” group (C2). Class 3 accounted for 30.3 and 20.6%, respectively, and was named the “high vividness” group (C3).

**TABLE 7:** Model fit of the latent profile models.

Number of profiles	AIC	BIC	aBIC	LMR (*p*)	BLRT (*p*)	Entropy	Class proportions
**PIT-P**							
2	36316.809	36462.063	36373.120	<0.001	<0.001	0.798	0.462/0.538
3	35776.545	35973.676	35852.967	<0.001	<0.001	0.768	0.229/0.469/0.302
4	35626.020	35875.027	35722.553	0.022	<0.001	0.763	0.350/0.110/0.226/0.314
5	35483.852	35784.736	35600.497	0.009	<0.001	0.810	0.226/0.057/0.339/0.292/0.086
**PIT-N**							
2	45788.514	45964.894	45856.891	<0.001	<0.001	0.820	0.515/0.485
3	45124.751	45363.383	45217.262	<0.001	<0.001	0.794	0.323/0.471/0.206
4	44898.027	45198.911	45014.672	0.012	<0.001	0.843	0.382/0.168/0.313/0.137
5	44633.937	44997.073	44774.715	0.003	<0.001	0.811	0.277/0.184/0.301/0.113/0.125

**N* = *1,372. AIC*, *Akaike information criterion; BIC*, *Bayesian information criterion; aBIC*, *sample-size-adjusted BIC; LMR*, *Lo–Mendell–Rubin; BLRT*, *bootstrapped likelihood ratio test.**

### Measurement Invariance Across Gender and Age

According to the multigroup CFA fit indices, the model fit was acceptable (all CFIs were close to 0.90, RMSEA < 0.08, SRMR < 0.08). Specifically, MI across genders was examined by means of multigroup CFA; the ΔCFIs were all < 0.010, and the ΔRMSEAs were all < 0.015 (see Table 8). Therefore, gender does not affect subjective judgments of prospective imagery. Subsequently, MI across age was examined by means of multigroup CFA; the ΔCFIs were all < 0.010, and the ΔRMSEAs were all < 0.015 (see Table 8). In other words, age does not affect subjective judgments of prospective imagery.

**TABLE 8:** Measurement invariance: multigroup CFA fit indices across gender and age groups.

Model	χ ^2^	*df*	Δχ ^2^	Δ*df*	CFI	RMSEA	SRMR	ΔCFI	ΔRMSEA
**Gender**									
Model 1 (configural invariance)	1318.17	338	–	–	0.871	0.066	0.062	–	–
Model 2 (metric invariance)	1335.69	356	17.52	18	0.871	0.064	0.063	0.000	−0.002
Model 3 (scalar invariance)	1430.80	374	95.11	18	0.861	0.065	0.064	−0.010	0.001
Model 4 (strict factorial invariance)	1463.50	394	32.70	20	0.859	0.064	0.066	−0.001	−0.001
**Age**									
Model 1 (configural invariance)	1333.05	338	–	–	0.870	0.067	0.061	–	–
Model 2 (metric invariance)	1353.87	356	20.82	18	0.870	0.065	0.063	0.000	−0.002
Model 3 (scalar invariance)	1421.21	374	67.34	18	0.864	0.065	0.064	−0.006	0.000
Model 4 (strict factorial invariance)	1506.55	394	85.34	20	0.855	0.065	0.069	−0.009	0.000

## Discussion

We aimed to develop a Chinese version of the PIT and to ensure that its psychometric properties were consistent with previous studies. The contributions of this study are threefold: first, we developed a native tool for measuring prospective imagery; second, we verified previous results; and third, we showed that the tool has significance as a reference for the diagnosis of clinical depression.

In this paper, the PIT was introduced to the mainland Chinese population. The structure of the revised Chinese version of the PIT scale was basically in line with the original research (Stöber, 2000). The original scale was divided into positive images (10 items) and negative images (10 items). EFA in this study showed that Item 18, belonging to positive dimensions, was classified as a negative dimension in this study. One reason for the inconsistency of the item “Your mind will be very alert and ‘on the ball”’ with the original scale may be that the meaning of “alert” differs among cultural backgrounds, leading to misunderstanding of the connotation of the item by domestic subjects. Another possibility is that individuals have different degrees of uncomfortable physical and mental experiences under stress (Dickerson et al., 2004). Participants are more likely to experience the discomfort of the stress state evoked by experiencing mental imagery. Thus, they tend to attach a negative meaning to it.

EFA showed that the structure of the Chinese version of the PIT included two stability factors of negative images (11 items) and positive images (9 items). Moreover, CFA determined that the goodness-of-fit indices of the two-factor model of the Chinese version of the PIT was acceptable.

The test-retest correlation coefficients in the current sample were good. Although the interval between baseline and follow-up was 2 months, the test-retest reliability was still statistically significant and approximated the internal reliability coefficient. The good correlation found in the present study supports this claim. The internal consistency coefficients of the total, positive, and negative scales were 0.84, 0.81, and 0.83, respectively. The retest reliability values after 2 months were 0.89, 0.78, and 0.89 at a significance level of *p* < 0.01, indicating high stability across time and good measurement requirements.

Stöber (2000) showed that only anxiety (but not depression) was related to enhanced imagery of future negative events. However, our study showed that positive imagery was negatively correlated with depression (*r* = −0.30, *p* < 0.01) and anxiety (*r* = −0.36, *p* < 0.01), while negative imagery was positively correlated with depression (*r* = 0.31, *p* < 0.01) and anxiety (*r* = 0.30, *p* < 0.01). Both findings have potentially important implications for research on anxiety and depression.

According to Šimundić (2009), AUC is generally used in ROC analysis to reflect the diagnostic performance of an evaluation tool. Positive prospective imagery may be significant as a reference for depression. A cut-off point of 19 provided optimum diagnostic accuracy against the BDI-II.

LPA revealed a three-class pattern. Three groups in two subscales were labeled “low vividness” (22.9 and 32.3% of the sample, respectively), “moderate vividness” (46.9 and 47.1% of the sample, respectively), and “high vividness” (30.3 and 20.6% of the sample, respectively) groups.

MI was mainly assessed in this study by analyzing and comparing four models: (1) configural invariance (equality of factor structure), (2) metric invariance (equality of factor structure and loadings), (3) scalar invariance (equality of factor structure, loadings, and intercepts), and (4) strict factorial invariance (equality of factor structure, loadings, intercepts, and unique variances). Furthermore, the MI showed that the PIT can be used among different age and gender groups.

### Limitations and Future Research Directions

There were several limitations of this study. First, the high proportion of women in our sample may not be representative of the community and may limit the generalizability of the results. Further research should validate the Chinese PIT by expanding the sample to a wider population. Second, it is uncertain whether participants actually followed the instructions to imagine positive or negative events and, if so, whether the contents of their prospective imagery were truly positive or negative. Finally, the present findings await replication with clinical participants.

## Conclusion

In summary, the structural validity, Cronbach’s α, and criterion validity of the Chinese PIT were verified, indicating that the scale’s reliability and validity had suitable adaptability under different sampling methods and different subpopulation conditions and that the scale had good ecological validity. The Chinese PIT is a reliable and valid instrument for assessing prospective imagery and, to some extent, depression.

## Data Availability Statement

The raw data supporting the conclusions of this article will be made available by the authors, without undue reservation, to any qualified researcher.

## Ethics Statement

The studies involving human participants were reviewed and approved by the Morals & Ethics Committee of the School of Psychology, Jiangxi Normal University (Nanchang, China). Written informed consent to participate in this study was provided by the participants’ legal guardian/next of kin.

## Author Contributions

ML and DP designed the research. ML, YC, XY, DP, XW, and BY performed the research. YC analyzed the data and wrote the manuscript. ML, XY, XW, and BY modified the manuscript. All authors read and approved the final manuscript.

## Conflict of Interest

The authors declare that the research was conducted in the absence of any commercial or financial relationships that could be construed as a potential conflict of interest.

## References

[B1] Beck A. T. Steer R. A. Ball R. Ranieri W. F. (1996). Comparison of beck depression inventories-IA and-II in psychiatric outpatients. *J. Pers. Assess.* 67 588–597. 10.1207/s15327752jpa6703_13 8991972

[B2] Blackwell S. E. Rius-Ottenheim N. Schulte-van Maaren Y. W. M. Carlier I. V. E. Middelkoop V. D. Zitman F. G. (2013). Optimism and mental imagery: a possible cognitive marker to promote well-being? *Psychol. Rev.* 206 56–61. 10.1016/j.psychres.2012.09.047 23084598 PMC3605581

[B3] Brewin C. R. Gregory J. D. Lipton M. Burgess N. (2010). Intrusive images in psychological disorders: characteristics, neural mechanisms, and treatment implications. *Psychol. Rev.* 117 210–232. 10.1037/a0018113 20063969 PMC2834572

[B4] Burnett Heyes S. Lau J. Y. F. Holmes E. A. (2013). Mental imagery, emotion and psychopathology across child and adolescent development. *Dev. Cogn. Neurosci.* 5 119–133. 10.1016/j.dcn.2013.02.004 23523985 PMC6987813

[B5] Cicchetti D. (1994). Guidelines, criteria, and rules of thumb for evaluating normed and standardized assessment instruments in psychology. *Psychol. Assess.* 6 284–290. 10.1037/1040-3590.6.4.284

[B6] Clark S. L. (2010). *Mixture Modeling With Behavioral Data.* Doctoral dissertation, California, CA: University of California.

[B7] Counsell A. Cribbie R. A. Flora D. B. (2020). Evaluating equivalence testing methods for measurement invariance. *Multivariate. Behav. Res.* 55 312–328. 10.1080/00273171.2019.1633617 31389729

[B8] Crane C. Shah D. Barnhofer T. Holmes E. A. (2012). Suicidal imagery in a previously depressed community sample. *Clin. Psychol. Psychother.* 19 57–69. 10.1002/cpp.741 21254309 PMC4615615

[B9] Dickerson S. S. Gruenewald T. L. Kemeny M. E. (2004). When the social self is threatened: shame, physiology, and health. *J. Pers.* 72 1191–1216. 10.1111/j.1467-6494.2004.00295.x 15509281

[B10] Finch J. F. West S. G. (1997). The investigation of personality structure: statistical models. *J. Res. Pers.* 31 439–485. 10.1006/jrpe.1997.2194

[B11] Finch W. H. French B. F. (2018). A simulation investigation of the performance of invariance assessment using equivalence testing procedures. *Struct. Equ. Modeling.* 25 673–686. 10.1080/10705511.2018.1431781

[B12] Gadassi Polack R. Tran T. B. Joormann J. (2020). “What has been is what will be”? Autobiographical memory and prediction of future events in depression. *Cogn. Emot.* 34 1044–1051. 10.1080/02699931.2019.1710467 31905320 PMC8695454

[B13] Green D. M. (1989). *Signal Detection Theory and Psychophysics*. Los Altos, CA: Peninsula Publishing.

[B14] Holmes E. A. Crane C. Fennell M. J. V. Williams J. M. G. (2007). Imagery about suicide in depression—flash-forwards? *J. Behav. Ther. Exp. Psychiatry.* 38 423–434. 10.1016/j.jbtep.2007.10.004 18037390 PMC2808471

[B15] Holmes E. A. Deeprose C. Fairburna C. G. WallaceHadrill S. M. A. Bonsall M. B. Geddes J. R. (2011). Mood stability versus mood instability in bipolar disorder: a possible role for emotional mental imagery. *Behav. Res. Ther.* 49 707–713. 10.1016/j.brat.2011.06.008 21798515 PMC3176902

[B16] Holmes E. A. Lang T. J. Moulds M. L. Steele A. M. (2008). Prospective and positive mental imagery deficits in dysphoria. *Behav. Res. Ther.* 46 976–981. 10.1016/j.brat.2008.04.009 18538304

[B17] Holmes E. A. Mathews A. (2010). Mental imagery in emotion and emotional disorders. *Clin. Psychol. Rev.* 30 349–362. 10.1016/j.cpr.2010.01.001 20116915

[B18] Kline R. B. (2010). *Principals and Practice of Structural Equation Modeling*, 3rd edn. New York, NY: Guilford Press.

[B19] Kosslyn S. M. Ganis G. Thompson W. L. (2001). Neural foundations of imagery. *Nat. Rev. Neurosci.* 2 635–642. 10.1038/35090055 11533731

[B20] Kosslyn S. M. Thompson W. L. Ganis G. (2006). *The Case for Mental Imagery*. New York, NY: Oxford University Press, Inc.

[B21] Liang B. C. Cherian J. Liu Y. L. (2010). Concrete thinking or ideographic language: which is the reason for Chinese people’s higher imagery-generation abilities? *Int. J. Consum. Stud.* 34 52–60. 10.1111/j.1470-6431.2009.00828.x

[B22] Liang B. C. Kale S. H. (2012). Cultural differences in imagery generation: the influence of abstract versus concrete thinking. *J. Bus. Res.* 65 333–339. 10.1016/j.jbusres.2011.04.010

[B23] MacLeod A. K. Byrne A. (1996). Anxiety, depression, and the anticipation of future positive and negative experiences. *J. Abnorm. Psychol.* 105 286–289. 10.1037/0021-843x.105.2.286 8723011

[B24] Morina N. Deeprose C. Pusowski C. Schmid M. Holmes E. A. (2011). Prospective mental imagery in patients with major depressive disorder or anxiety disorders. *J. Anxiety. Disord.* 25 1032–1037. 10.1016/j.janxdis.2011.06.012 21783339 PMC3389342

[B25] Mulaik S. A. James L. R. Alstine J. V. Bennett N. Lind S. Stilwell C. D. (1989). Evaluation of goodness-of-fit indices for structural equation models. *Psychol. Bull.* 105 430–445. 10.1037/0033-2909.105.3.430

[B26] Muthén L. K. Muthén B. O. (1998). *Mplus User’s Guide*, 7th Edn. Los Angeles, CA: Muthén & Muthén.

[B27] Nylund K. L. Asparouhov T. Muthén B. O. (2007). Deciding on the number of classes in latent class analysis and growth mixture modeling: a monte carlo simulation study. *Struct. Equ. Modeling.* 14 535–569. 10.1080/10705510701575396

[B28] Rief W. Glombiewski J. A. Gollwitzer M. Schubö A. Schwarting R. Thorwart A. (2015). Expectancies as core features of mental disorders. *Curr. Opin. Psychiatr.* 28 378–385. 10.1097/yco.0000000000000184 26164612

[B29] Shek D. T. L. (1988). Reliability and factorial structure of the Chinese version of the State-Trait Anxiety Inventory. *J. Psychopathol. Behav. Assess.* 10 303–317. 10.1007/bf00960624

[B30] Shek D. T. L. (1993). The Chinese version of the State-Trait Anxiety Inventory: its relationship to different measures of psychological well-being. *J. Clin. Psychol.* 49 349–358. 10.1002/1097-4679(199305)49:3<349::aid-jclp2270490308>3.0.co;2-j 8315037

[B31] Šimundić A. (2009). Measures of diagnostic accuracy: basic definitions. *Med. Biol. Sci.* 19 203–211. PMC4975285 27683318

[B32] Spielberger C. D. Gorsuch R. L. Lushene R. E. (1970). Manual for the State-Trait Anxiety Inventory. Palo Alto, CA: Consulting Psychologists Press

[B33] Stöber J. (2000). Prospective cognitions in anxiety and depression: replication and methodological extension. *Cogn. Emot.* 14 725–729. 10.1080/02699930050117693

[B34] Wang Z. Yuan C. M. Huang J. Li Z. Z. Chen J. Zhang H. Y. (2011). Reliability and validity of the Chinese version of Beck Depression Inventory-II among depression patients. *Chin. Ment. Health. J.* 25 476–480.

[B35] Werner-Seidler A. Moulds M. L. (2011). Autobiographical memory characteristics in depression vulnerability: formerly depressed individuals recall less vivid positive memories. *Cogn. Emot.* 25 1087–1103. 10.1080/02699931.2010.531007 21895571

[B36] Yoon H. Schwarz I. Nippold M. A. (2016). Comparing proverb comprehension in Korean and American youth. *Speech Lang Hear*. 19 161–170. 10.1080/2050571x.2016.1164938

