# Supplementary material for: Measuring Prospective Imagery: Psychometric Properties of the Chinese Version of the Prospective Imagery Task
Source: Front Psychol. 2021 May 25;12:645127. doi: 10.3389/fpsyg.2021.645127 (PMC8185034; doi:10.3389/fpsyg.2021.645127)
Supplement: Supplementary file 1 [file Table_1.DOCX]

Read each statement and then imagine each future scenario happening to you personally. When you have a mental image of the scenario happening to you, please rate the vividness of each image on a 5-point scale (Vividness refers to how clear and detailed the image is).

|  |  | no image at all | vague and dim | unclear but recognizable | moderately vivid | very vivid |
| --- | --- | --- | --- | --- | --- | --- |
| 1 | You will have a serious disagreement with a good friend | 1 | 2 | 3 | 4 | 5 |
| 2 | People will admire you | 1 | 2 | 3 | 4 | 5 |
| 3 | You will have health problems | 1 | 2 | 3 | 4 | 5 |
| 4 | You will make a decision you regret | 1 | 2 | 3 | 4 | 5 |
| 5 | You will feel misunderstood | 1 | 2 | 3 | 4 | 5 |
| 6 | You will have lots of energy and enthusiasm | 1 | 2 | 3 | 4 | 5 |
| 7 | You will do well on your course | 1 | 2 | 3 | 4 | 5 |
| 8 | You will get the blame for things going wrong | 1 | 2 | 3 | 4 | 5 |
| 9 | You will achieve the things you set out to do | 1 | 2 | 3 | 4 | 5 |
| 10 | You will be the victim of crime | 1 | 2 | 3 | 4 | 5 |
| 11 | Someone close to you will reject you | 1 | 2 | 3 | 4 | 5 |
| 12 | Things won’t work out as you had hoped | 1 | 2 | 3 | 4 | 5 |
| 13 | People will dislike you | 1 | 2 | 3 | 4 | 5 |
| 14 | You will be very fit and healthy | 1 | 2 | 3 | 4 | 5 |
| 15 | People will find you dull and boring | 1 | 2 | 3 | 4 | 5 |
| 16 | You will have lots of good times with friends | 1 | 2 | 3 | 4 | 5 |
| 17 | You will be able to cope easily with pressure | 1 | 2 | 3 | 4 | 5 |
| 18 | You mind will be very alert and “on the ball” | 1 | 2 | 3 | 4 | 5 |
| 19 | You will make good and lasting friendships | 1 | 2 | 3 | 4 | 5 |
| 20 | People you meet will like you | 1 | 2 | 3 | 4 | 5 |

|  | 根本  没有  图像 | 模糊 | 模糊  但可以  识别 | 生动 | 非常  生动 |
| --- | --- | --- | --- | --- | --- |
| 1. 你将会和一个好朋友发生严重的争执 | 1 | 2 | 3 | 4 | 5 |
| 1. 人们将会赞扬你 | 1 | 2 | 3 | 4 | 5 |
| 1. 你将会有健康问题 | 1 | 2 | 3 | 4 | 5 |
| 1. 你将会做出一个让自己后悔的决定 | 1 | 2 | 3 | 4 | 5 |
| 1. 你将会感觉被误解 | 1 | 2 | 3 | 4 | 5 |
| 1. 你将会有很多精力和热情 | 1 | 2 | 3 | 4 | 5 |
| 1. 你将会在课程/工作上取得好成绩 | 1 | 2 | 3 | 4 | 5 |
| 1. 你将会因为事情出错而受到责备 | 1 | 2 | 3 | 4 | 5 |
| 1. 你将实现你想要做的事情 | 1 | 2 | 3 | 4 | 5 |
| 1. 你将成为犯罪行为的受害者 | 1 | 2 | 3 | 4 | 5 |
| 1. 你身边的人将会拒绝你 | 1 | 2 | 3 | 4 | 5 |
| 1. 事情不会如你所希望的那样顺利 | 1 | 2 | 3 | 4 | 5 |
| 1. 人们将会不喜欢你 | 1 | 2 | 3 | 4 | 5 |
| 1. 你将非常健康 | 1 | 2 | 3 | 4 | 5 |
| 1. 人们将会发现你既沉闷又无聊 | 1 | 2 | 3 | 4 | 5 |
| 1. 你将与朋友一起度过美好时光 | 1 | 2 | 3 | 4 | 5 |
| 1. 你将能轻松应对压力 | 1 | 2 | 3 | 4 | 5 |
| 1. 你将会变得非常警觉 | 1 | 2 | 3 | 4 | 5 |
| 1. 你将会建立良好而持久的友谊 | 1 | 2 | 3 | 4 | 5 |
| 1. 你遇到的人将会喜欢你 | 1 | 2 | 3 | 4 | 5 |
